# Thermal Degradation Studies of Poly(2-ethyl hexyl acrylate) in the Presence of Nematic Liquid Crystals

**DOI:** 10.3390/polym15193934

**Published:** 2023-09-29

**Authors:** Amina Bouriche, Lamia Alachaher-Bedjaoui, Ana Barrera, Jean-Noël Staelens, Ulrich Maschke

**Affiliations:** 1 Laboratoire de Recherche sur les Macromolécules (LRM), Faculté des Sciences, Université AbouBekr Belkaïd de Tlemcen (UABB), BP 119, Tlemcen 13000, Algeria; 2 Unité Matériaux et Transformations (UMET), UMR 8207, Université Lille, CNRS, INRAE, Centrale Lille, 59000 Lille, France

**Keywords:** polymer, liquid crystal, thermal stability, thermogravimetric analysis, non-isothermal method

## Abstract

The thermal degradation behavior of Poly(2-ethyl hexyl hcrylate) (Poly(2-EHA)), blended with a commercially available nematic liquid crystal (LC) mixture, was investigated by thermal gravimetric analysis (TGA). Different heating rates, ranging from 5 to 200 °C/min, were applied under an inert atmosphere. Based on the TGA results, activation energies (*E_α_*) at different conversion rates (*α*) were determined using three integral isoconversion methods: Flynn-Wall-Ozawa (FWO), Tang, and Kissinger-Akahira-Sunose (KAS). It can be noticed that the global evolution of these activation energies was the same for the three models. The coefficient of determination *R*^2^ presented values generally higher than 0.97. Using these models, the *E_α_* value for the LC remains constant at 64 kJ/mol for all conversions rates. For the polymer Poly(2-EHA), applying the Tang and FWO models, the activation energy presents a variation ranging from 80 kJ/mol, for conversion *α* = 0.1, to 170 kJ/mol, for *α* = 0.9. For the third model (KAS), this energy varies between 80 and 220 kJ/mol in the same range of *α*.

## 1. Introduction

Polymer dispersed liquid crystal (PDLC) films remain an important class of compo-site materials consisting of phase-separated micro-sized LC domains embedded in a con-tinuous polymer matrix [1,2]. Recently, PDLC films have been widely investigated due to the electrically responsive and birefringent properties of LCs and the excellent properties of selected polymers such as good mechanical strength, flexibility and easy processability [3]. Inspired by the use of PDLC materials in display technologies, research on these mate-rials has grown rapidly in the last decades and now extends beyond smart windows [4], displays [5], including diffuse [6], antipeeping and quantum dots (QDs) [7] devices, com-ponents of organic light-emitting diodes (OLEDs) [8], field-effect transistors (FETs) [9], energy storage [10] and solar energy harvesting [11].

Because polymeric materials are often used outdoors for long periods of time, they can be subject to degradation effects caused by heat, sunlight, atmospheric oxygen, mois-ture and stress. As a result, their chemical and physical properties deteriorate and the life of the material is limited. Therefore, any polymeric material to be used in outdoor applica-tions must have excellent resistance to all environmental conditions. The thermal degra-dation behavior of polymers affects the final properties, such as the upper temperature limit of use and dimensional stability. The reliability of polymer materials can be im-proved and the competitiveness of a product increased by evaluating the durability and estimating the service life [12].

Few techniques are available to evaluate the durability of PDLC materials. Thermogravimetric analysis (TGA) is commonly used to determine the thermal decomposition kinetics and thermal stability of polymers [13] and can be studied using single heating and multiple heating rate methods [14]. Model-free isoconversion methods [15,16] are the most reliable approaches for calculating activation energies of thermally activated reactions. A large number of isoconversion methods have been proposed, that allow the activation energy (*E*) of a process to be estimated as a function of the degree of conversion (*α*) [17]. Analysis of the dependence of the activation energy on *α* provides important clues about the degradation mechanism [18,19,20,21,22]. It is worth noting that when the activation energy is dependent on *α* [23], the differential method proposed by Friedman [24] gives values of the activation energy that differ from those obtained by integral iso-conversion methods such as Flynn-Wall-Ozawa (FWO) [25,26], Li-Tang [27] and Kissinger-Akahira-Sunose (KAS) [28].

Thermal degradation reactions of polyacrylates generally occur through various re-action pathways leading to peroxides, alcohols, carbon dioxide, etc. along with depoly-merized structures such as monomers [29,30,31]. In the presence of oxygen, the polymer degradation rate is usually higher than in an oxygen-free environment [32,33,34]. Hu et al. compared the thermal degradation of poly(butyl acrylate) (PBA) initiated by the combination of 3-caprolactam and thiols to initiate vinyl polymerization, and the novel system was found to be different from the traditional PBA initiated with azobisisobutyronitrile (AIBN). PBAs prepared with thiols have higher degradation temperatures and corre-sponding activation energies compared to those prepared with AIBN [35]. Indeed, PBA-thiols have an average activation energy of 170–212 kJ/mol, while that of PBA-AIBN was found to be 130 kJ/mol, using the KAS method. This energy varied between 155–182 kJ/mol for PBA-thiols and 113 kJ/mol for PBA-AIBN when the FWO method was used.

Qian et al. investigated different compositions of poly(2-ethylhexylacrylate/α-methylstyrene) (2-EHA/MeSt) copolymers [36]. As the content of α-MeSt units increased from 0 wt-% to 20 wt-%, the degradation activation energy of the copolymers increased from 127.1 to 148.5 kJ/mol. The FWO method showed that the value of the degradation activation energy of the α-MeSt-20 sample was higher than that of α-MeSt-0 at each conversion, and with the progress of the degradation reaction, the value of the degradation activation energy of α-MeSt-20 gradually approached that of α-MeSt-0. Ors and la Perrière [37] proposed a degradation mechanism of poly(isobornylacrylate) (poly(IBOA)) leading to the formation of both camphene and a nortricyclic species. This two-step degradation mechanism was also observed by Matsumoto et al. [38]. A thermal degradation study of a polymer/LC system composed of poly(ethylene-co-methylacrylic acid) (PEMA) and 4-(3-hydroxypropoxy)-4′-cyanobiphenyl (H3CB) was carried out by Han et al. [39]. The temperature of maximum decomposition rate was observed to be 318 °C for H3CB and 492 °C for PEMA. The process of thermal decomposition under inert atmosphere involves chain scission effects, and interaction occurred between PEMA and H3CB. A two-step decomposition process occurred in nitrogen atmosphere.

Edina et al. studied the synthesis and characterization of nano-encapsulated LCs in a polymer (nanoELCP) obtained by soap-free emulsion polymerization of the system sty-rene (ST) and acrylic acid (AA) in the presence of the LC E7 [40]. TGA analyses were per-formed to quantify the amount of E7 encapsulated in the polymer particles [41]. The prep-aration and experimental studies of phase transitions and morphology of new PDLC sys-tems based on Udel-3000 polysulfone (PSU) or its derivatives (chloromethylated PSU or PSU with a phosphaphenanthrene side substituent) and a phosphorus-containing LC polymer (LCP) were evaluated by Tachita et al. [42]. These researchers showed that the presence of the phosphorus atom in the structure of the LCP and also in the polymer matrix, based on phosphorylated PSU, improved the key properties such as flame retardancy, thermal stability, and so on.

Merah et al. synthesized Poly(IBOA-co-2-EHA) copolymers via free radical photo-polymerization/crosslinking reactions of IBOA and 2-EHA, in the presence of 1,6-hexanedioldiacrylate (HDDA) as crosslinking agent, to obtain chemically crosslinked polymer networks [43]. Several degradation processes were observed by TGA, especially that of the isobornylene group at low temperature, followed by the degradation of the carbon backbone at higher temperatures. Increasing IBOA content leads to a higher thermal stability of Poly (IBOA-co-2-EHA). The mass loss thermogram and its derivative of cross-linked poly(2-EHA) revealed that its thermal degradation takes place in a single step be-tween 593 and 693 K, corresponding to the decomposition of the carbon skeleton. This ob-servation has already been made by Haloi and Singha [44], by investigating the thermal degradation of poly(2-EHA)/nanocomposite clay in an interval from 597 and 687 K.

Although much research has been done on thermal effects related to physical proper-ties of polymers, not many studies are known to have investigated the kinetics of thermal decomposition and thermal stability of polymer/LC blends. In the present work, a model polymer/LC system was investigated by TGA using different heating rates. The system investigated here is composed of linear poly(2-EHA) and the eutectic nematic blend E7, by varying their composition. Three multiple heating rate methods, Li Tang, FWO and KAS, were applied to calculate the activation energies of thermal decomposition. The local linear integral isoconversion method (LL-INT) was chosen to evaluate the activation energy dependencies under non-isothermal conditions [45].

## 2. Materials and Methods

### 2.1. Materials

**Figure 1:**
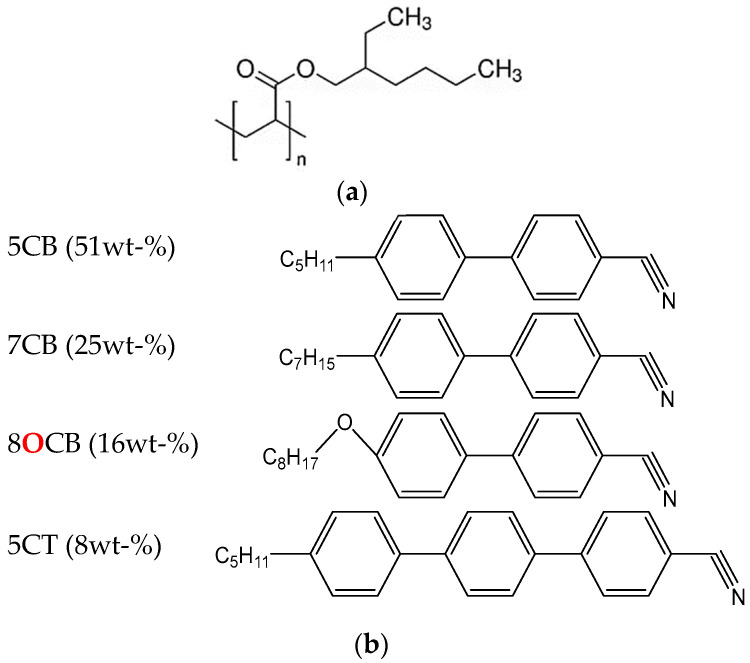
Chemical structures of (**a**) Poly 2-EHA and (**b**) E7.

Poly(2-EHA) (see Figure 1a) was supplied by Sigma Aldrich (Saint Quentin Fallavier, France). Its molecular weight and polydispersity (*M*_w_/*M*_n_) were determined by gel permeation chromatography (GPC), yielding *M*_w_ = 92.000 g/mol and *M*_w_/*M*_n_ = 3.

E7 represents a well-known nematic LC mixture (see Figure 1b), commercially available from Synthon GmbH (Wolfen, Germany). This eutectic mixture contains 51 wt-% of 4-cyano-4′-n-pentyl-biphenyl (5CB), 25 wt-% of 4-cyano-4′-n-heptyl-biphenyl (7CB), 16 wt-% of 4-cyano-4′-n-oxyoctyl-biphenyl (8OCB) and 8 wt-% of 4-cyano-4″-n-pentyl-p-biphenyl (5CT) [46]. E7 exhibits a single nematic-isotropic transition temperature (*T*_NI_) at +61 °C, and a glass transition temperature (*T*_g_) at −61 °C [47]. The ordinary (*n*_o_) and extraordinary (*n*_e_) refractive indices of E7 at *T* = 20 °C are given as *n*_o_ = 1.5183; *n*_e_ = 1.7378 (*λ* = 632.8 nm), leading to an optical birefringence of Δ*n* = *n*_e_ − *n*_o_ = 0.2195 [48]. The chemical structures of the components of E7 used in this study are shown in Figure 1b). The poly 2-EHA/E7 blends were prepared by a thermally induced phase separation process. *x* wt-% of E7 (*x* = 10, 20,…, 90) and (100 − *x*) wt-% of poly-2EHA were mixed together at room temperature for several hours. Detailed data on the evolution of the glass transition and the transition from the nematic + isotropic to the isotropic states of the poly-2-EHA/E7 mixtures with composition are given in reference [47]. Some optical data are also given [47].

### 2.2. Thermogravimetrical Analysis

TGA was performed on a Perkin Elmer Pyris 1 analyzer (Perkin Elmer, Waltham, MA, USA) with a mass resolution of 1 μg using HT platinum plates. Analysis of samples with an average weight of 8 mg was performed under a nitrogen atmosphere at a flow rate of 20 mL/min. The precise temperature was measured by a thermocouple in direct contact with the sample crucible. Thermogravimetric analysis of the polymer/LC samples was performed in dynamic mode using constant masses subjected to a series of heating rates *β* (5, 10, 20, 50, 100 and 200 °C/min), applying a temperature interval between 20 °C and 800 °C.

### 2.3. Gel permeation Chromatography

Gel permeation chromatography (GPC) analysis was performed at room temperature on a Waters Alliance e2695 system (Waters S.A.S., Saint-Quentin en Yvelines, France) using differential refractive index (Wyatt RI) and multi-angle light scattering (Wyatt MALS, *λ* (laser) = 670 nm) detectors. Three columns were coupled in series (Styragel HR1, Styragel HR3, Styragel HR4). Tetrahydrofuran (THF) was used as solvent (flow rate 1 mL/min) and calibration was performed with polystyrene (PS) standards from Polymer Laboratories. A fixed amount of 5 mg of the sample was dissolved in 5 mL of THF and filtered through a 0.20 µm filter to remove undissolved particles.

### 2.4. Polarized Optical Microscopy

Polarized Optical Microscopy (POM) experiments were performed using an Olympus BX41 equipped with a Linkam LTS350 heating/cooling stage and a TMS 94 temperature control unit. Specimens were placed between two microscope slides, heated until optically clear, and then slowly cooled to ambient temperature. The heating and cooling rates were 0.5 °C/min. The same heating and cooling procedures were repeated three times for each sample to minimize experimental uncertainty.

## 3. Kinetic Study of Degradation

In thermogravimetric measurements, the degree of decomposition (conversion) can be calculated as follows

(1) $$\alpha =\frac{m_{0}-m_{t}}{m_{0}-m_{f}}$$

where α represents the relative decomposition conversion [49]. $m_{0}$, $m_{f}$, and $m_{t}\;$ correspond to the masses of the sample ($m_{0}$, in the initial state; $m_{f}$, in the final state, $m_{t}$, at any given time *t*). α was set to 0 and 1 for the initial and final measurement data, respectively [49].

A typical model for a kinetic process can be represented as follows

(2) dα/dt=k f(α)

where dα/dt stands for the decomposition rate, k is a decomposition rate constant that can be expressed as α, and $f\left(\alpha \right)$ represents the function of *α* which depends on the decomposition mechanism. The relationship between reaction rate and extent of reaction can be generally expressed in the form

(3) $$d\alpha /dt=A\;exp(-E_{\alpha }/RT)\;f\left(\alpha \right)$$

where *A* is the pre-exponential factor (s^−1^), *E_α_* stands for the activation energy (J/mol), *R* represents the gas constant (8.314 J/mol K), and *T* corresponds to the temperature in Kelvin [50].

### 3.1. Tang Method

This method represents an integral method, using an approximation of the integral temperature as proposed by Tang et al. [51]

(4) $$\ln\left(\frac{\beta }{T_{\alpha }^{1.894661}}\right)=C_{T}\left(\alpha \right)-1.0014(\frac{E_{\alpha }}{{RT}_{\alpha i}})$$

where $C_{T}(\alpha )$ is a constant equal to $\left[\ln\frac{{AE}_{\alpha }}{g\left(\alpha \right)R}+3.6350-1.8946nE_{\alpha }\right]$. The index *i* denotes different heating rates. For each degree of conversion *α*, a corresponding *T_αi_* and a heating rate are used. *E_α_* is evaluated from the slope of the linear regression of the plot $\ln(\frac{\beta }{T_{\alpha i}^{1.894661}}$) as a function of 1/T [52].

### 3.2. Flynn-Wall-Ozawa Method

The FWO method is a model-free method developed by Flynn and Wall [53] as well as by Ozawa. FWO uses Doyle’s [54] equation for the approximation of the temperature integral. Considering the approximation $\ln p\left(x\right)=-5.331-1.052x$, Equation (3) can be converted into

(5) $$\ln\beta =C_{W}\left(\alpha \right)-1.052\frac{E_{\alpha }}{{RT}_{\alpha i}}$$

where *C_W_* (*α*) stands for a constant equal to $\ln\left[\frac{{AE}_{\alpha }}{g\left(\alpha \right)R}\right]-5.331.$ *E_α_* is evaluated from the slope of the linear regression of the plot *ln β* as a function of $1/T_{\alpha i}$ [55].

### 3.3. Kissinger-Akahira-Sunose Method

The method of KAS is also known as the generalized Kissinger method [56]. This isoconversional integral method, based on the Murray and White approximation, is given by

(6) $$ln\frac{\beta_{i}}{T_{\alpha i}^{2}}=C_{K}\left(\alpha \right)-\frac{E_{\alpha }}{{RT}_{\alpha i}}$$

where $C_{K}(\alpha )$ represents a constant equal to $ln\frac{AR}{E_{\alpha }g(\alpha )}$. *E_α_* can be calculated from the slope obtained by plotting $ln\frac{\beta_{i}}{T_{\alpha i}^{2}}$ as function of $1/T_{\alpha i}$ [57].

## 4. Results and Discussion

Thermogravimetric curves and their derivatives (DTG) have been used to evaluate the thermal stability of poly(2-EHA)/E7 blends. The area of the DTG peak is directly proportional to the mass loss of the material over the same temperature range, and the height of the (DTG) peak indicates the rate of mass loss at the corresponding temperature.

### 4.1. Liquid Crystal E7

**Figure 2:**
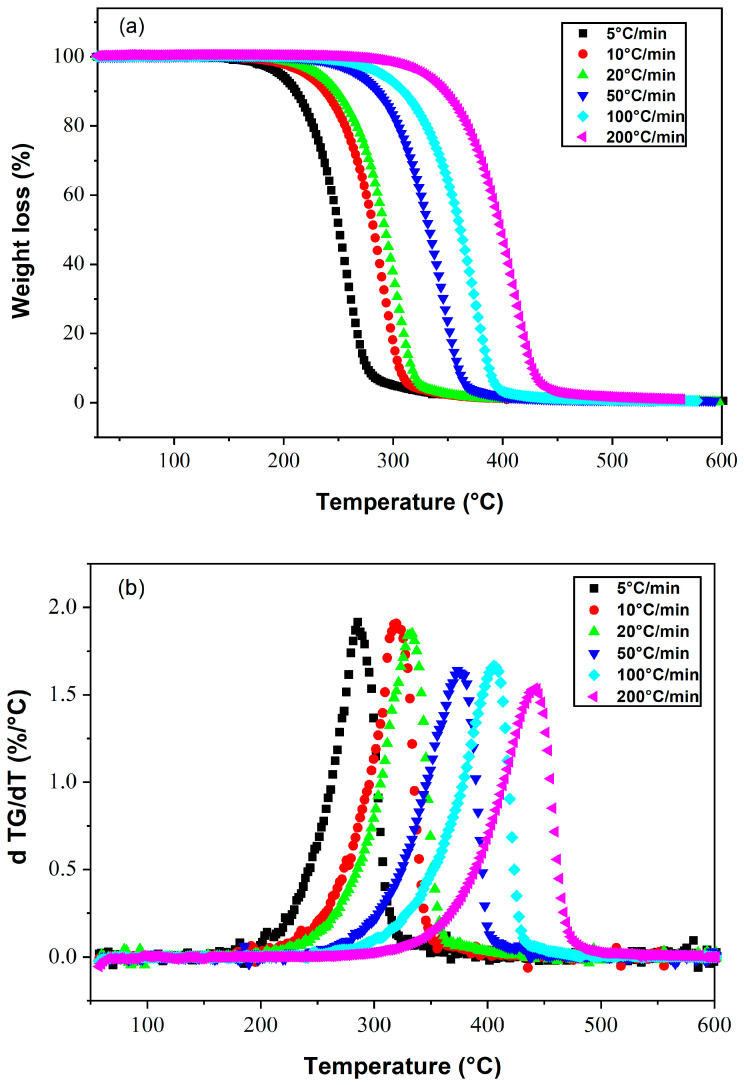
Thermograms presenting (**a**) the weight losses and (**b**) their derivatives as a function of temperature for the LC E7, at different heating rates.

The evolution of the mass loss (in %) and the rate of mass loss (DTA) during the decomposition of E7, obtained by applying different heating rates, are shown in Figure 2a and Figure 2b, respectively. This LC mixture remains thermally stable up to 180 °C at a heating rate of 5 °C/min. Total degradation was achieved around 300 °C. On the other hand, when a heating rate of 200 °C/min is applied, the range of thermal stability is extended up to 310 °C, and total degradation occurs at 450 °C. It can be concluded that the temperature corresponding to the maximum of the mass derivative increases with the heating rate, together with the expansion of the temperature range of thermal stability.

### 4.2. Poly(2-Ethylhexylacrylate)

**Figure 3:**
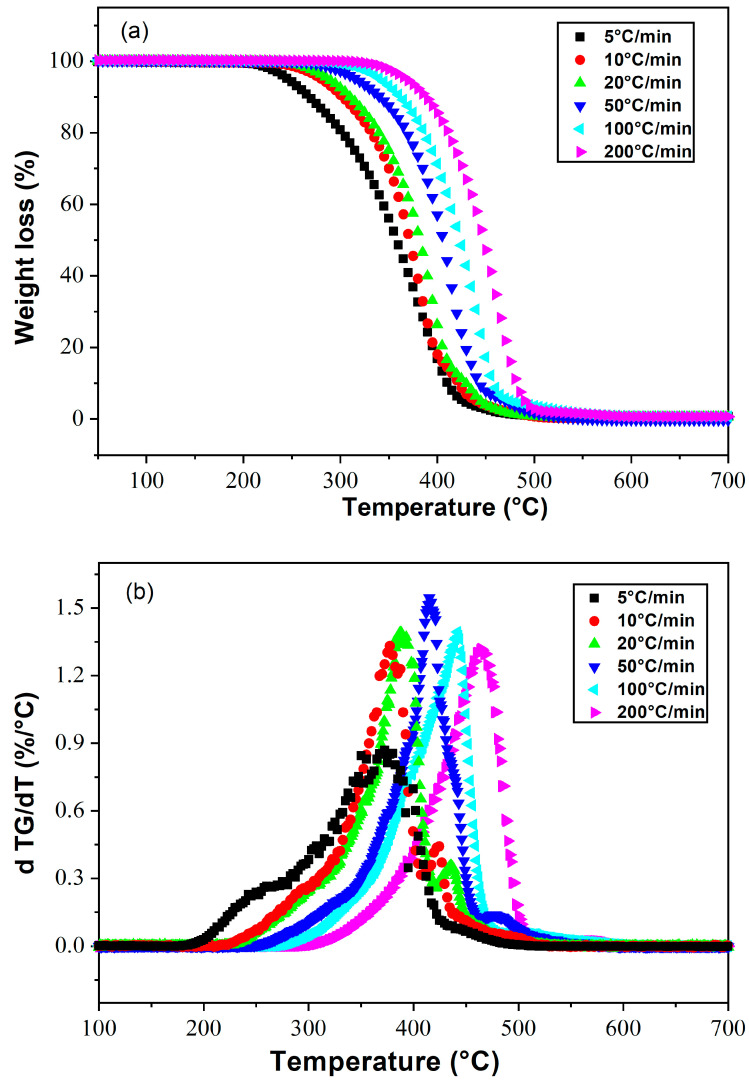
Thermograms presenting (**a**) the weight losses and (**b**) their derivatives as a function of temperature for Poly(2-EHA), at different heating rates.

Thermal degradation of polyacrylates in air occurs by peroxide formation, radical reactions with oxygen leading to depolymerization, formation of alcohols, carbon dioxide, etc. In the absence of oxygen, polyacrylates degrade by rearrangements leading to decarboxylation and formation of monomers and alcohols [58]. Figure 3a shows the degradation kinetics of poly(2-EHA) at different heating rates, and Figure 3b shows the derivatives of mass loss as a function of temperature. A thermal stabilization was observed between 25 °C and 250 °C for the lowest heating rate (5 °C/min), which broadened as the heating rate increased. The degradation temperature of poly(2-EHA) was determined at the peak maximum as shown in Figure 3b. The appearance of two shoulders was noticed, the first appeared in the temperature range from 150 to 370 °C and the second in the interval from 400 to 450 °C. The latter disappeared by increasing the heating rate. This phenomenon can be related to the mobility of the chain segments of the linear poly(2-EHA) and was not observed in the case of crosslinked polymers, where the zone of thermal stability increases with the increase of the crosslinking rate [59].

A qualitative review of the thermal degradation reactions occurring on linear poly(2-EHA) has been performed by Grassie et al. [60]. In particular, the thermal volatilization analysis of poly(2-EHA) leads to a two-step degradation mechanism (see Figure 6 of [60]). The molecular origin of such double degradation phenomena has been described by Aliev et al. [61] who studied the thermal stability of poly(alkyl acrylates). In fact, the degradation proceeds in two stages, corresponding to two mechanisms of polymer depolymerization with random chain initiation at the ends of the molecule and inside the molecule.

### 4.3. Poly(2-EHA)/E7 Blends

**Figure 4:**
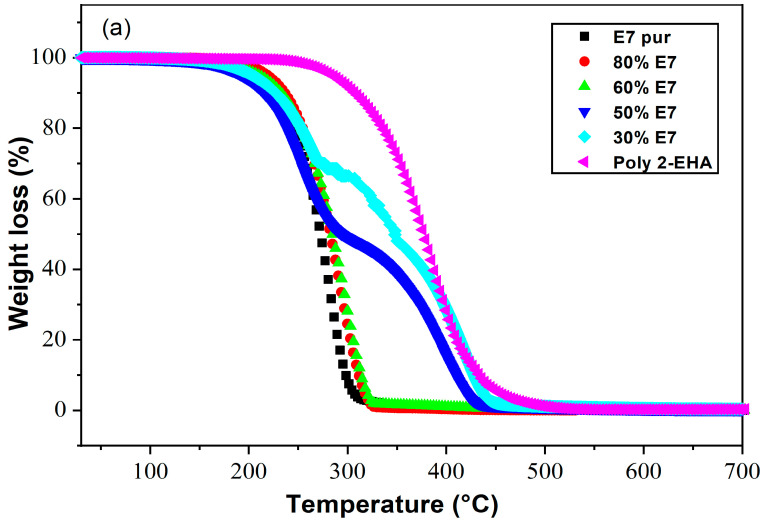

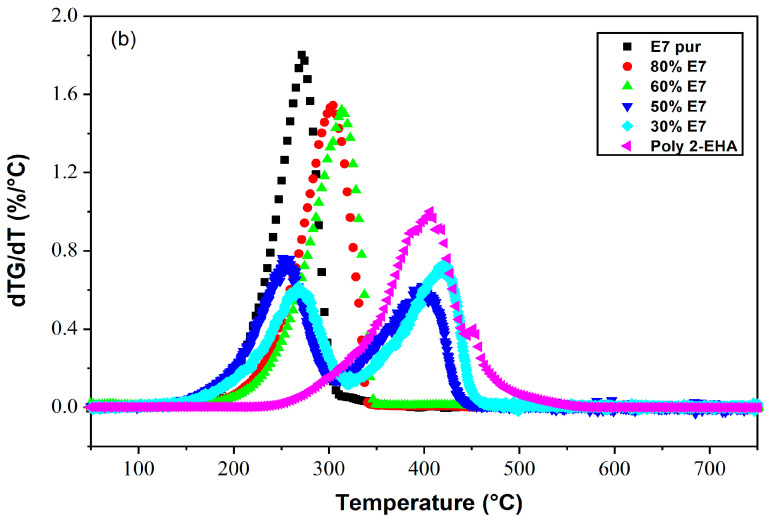
Thermograms presenting (**a**) the weight losses and (**b**) their derivatives as functions of temperature and composition for Poly(2-EHA)/E7 mixtures (heating rate: 10 °C/min).

Figure 4 shows the loss of mass (Figure 4a) and its derivatives (Figure 4b) as a function of temperature of Poly(2-EHA)/E7 mixtures for different sample compositions, for a heating rate of 10 °C/min. For blends containing high concentrations of E7 (above 60 wt-%), the TGA results showed a thermal behavior similar to that of pure E7. In this case, a single degradation temperature corresponding to E7 was observed, which increased with the concentration of LC. For lower E7 content (below 60 wt-%), two degradation temperatures were observed, one corresponding to Poly(2-EHA), and the other to the LC. For polymer-rich mixtures, a slight variation in the degradation temperatures of E7 and polymer was observed.

These observations can be explained by the evolution of the phase behavior, i.e., the morphologies, as a function of sample composition. As will be shown in the next Section 4.4, at higher E7 concentrations (above 60 wt-% E7) coalescence phenomena occur, resulting in large LC droplets that dominate the sample morphology, so that only a single degradation temperature was observed. On the other hand, as shown in Figure 4, at lower E7 concentrations (below 60 wt-% E7), separated small LC domains are present, resulting in the appearance of two degradation temperatures.

The results obtained by TGA were rationalized by isoconversion methods to calculate the kinetic parameters. In particular, *E_α_* was obtained using theoretical methods developed by Tang, FWO and KAS.

### 4.4. Observation of Sample Morphology of Poly(2-EHA)/E7 Blends

**Figure 5:**
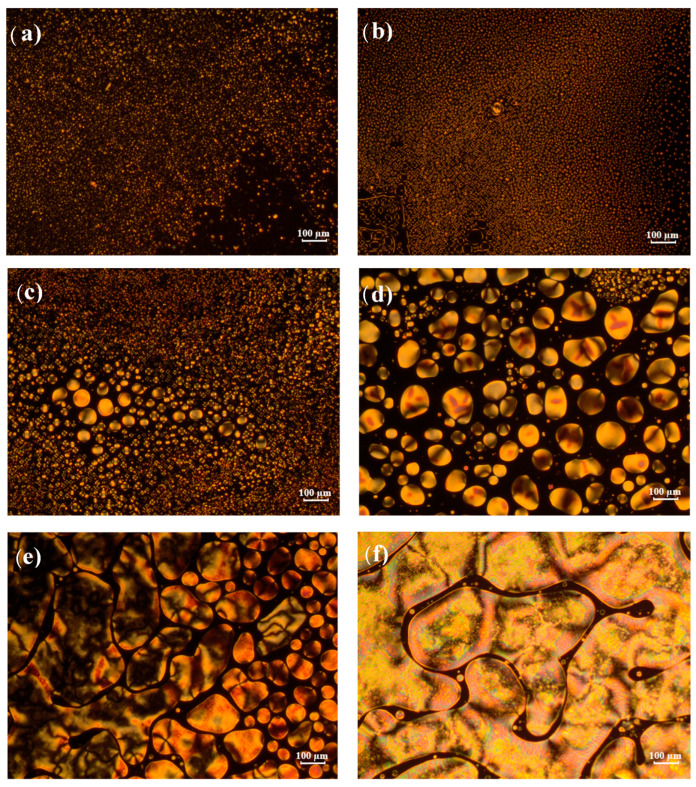
Texture of Poly-2 EHA/E7 samples at different concentrations observed at 58 °C in the nematic + isotropic state: (**a**) 30 wt-% E7, (**b**) 40 wt-% E7, (**c**) 60 wt-% E7, (**d**) 70 wt-% E7, (**e**) 80 wt-% E7, and (**f**) 90 wt-% E7.

Analysis of the morphology of p-2EHA/E7 mixtures with POM makes it possible to observe the variation of the texture of the LCs as a function of temperature and sample composition. Figure 5 shows some POM images of such sample morphologies of these mixtures with different concentrations of E7, taken at 58 °C with crossed polarizer/analyzer. This temperature is close to the nematic-isotropic transition temperature. For mixtures with low E7 content (30 wt-% and 40 wt-%, Figure 5a,b), the LC was already phase-separated from the polymer and presented small but distinguishable droplets. The droplets decrease in number and increase in size for blends above 40 wt-% E7 (Figure 5c–f). Coalescence phenomena of the LC droplets occur and are clearly visible on the POM scale, especially for mixtures above 70 wt-% E7 (Figure 5d). At 80 and 90 wt-% E7 (Figure 5e,f), coalescence effects lead to large scale patterns with dimensions of several hundreds of µm.

### 4.5. Determination of Activation Energy

**Figure 6:**
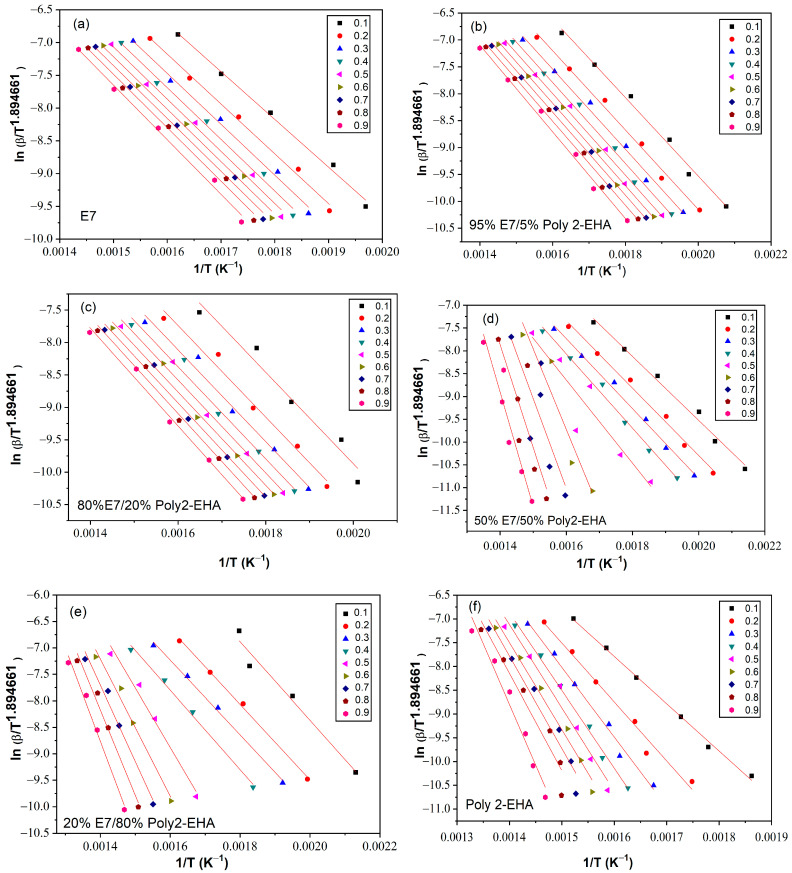
*Ln*(*β*/*T*^1.894661^) as function of 1/*T* for Poly(2-EHA)/E7 mixtures, according to the conversion *α*, using the approach of Tang et al. (**a**) 100 wt-% E7, (**b**) 95 wt-% E7, (**c**) 80 wt-% E7, (**d**) 50 wt-% E7, (**e**) 20 wt-% E7 and (**f**) Poly(2-EHA).

**Figure 7:**
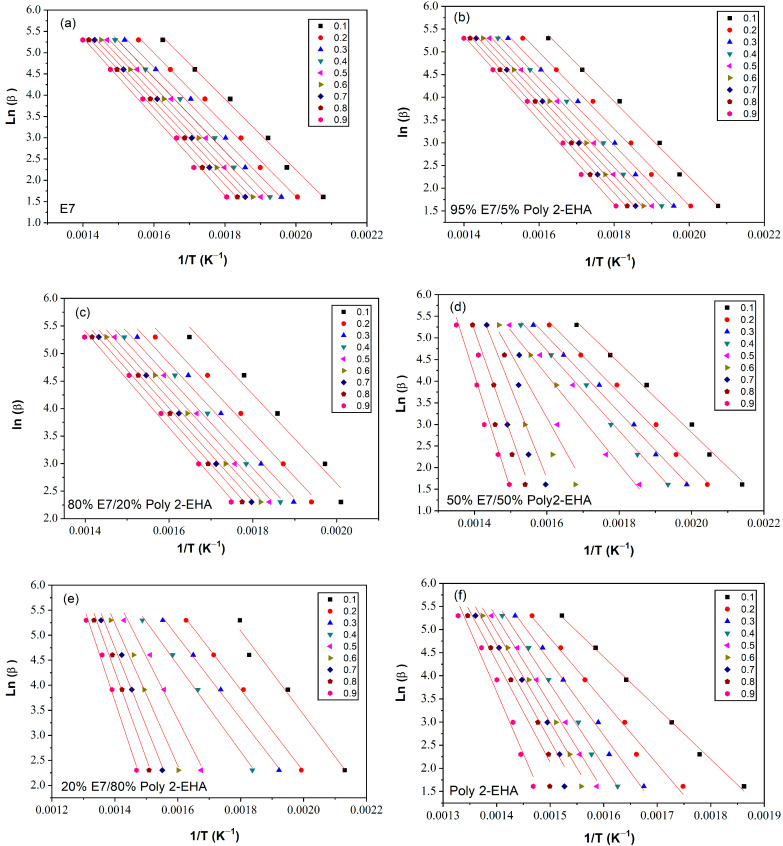
*Ln*(*β*) as function of 1/*T* for Poly(2-EHA)/E7 mixtures, according to the conversion *α*, applying the approach of FWO et al. (**a**) 100 wt-% E7, (**b**) 95 wt-% E7, (**c**) 80 wt-% E7, (**d**) 50 wt-% E7, (**e**) 20 wt-% E7 and (**f**) Poly(2-EHA).

**Figure 8:**
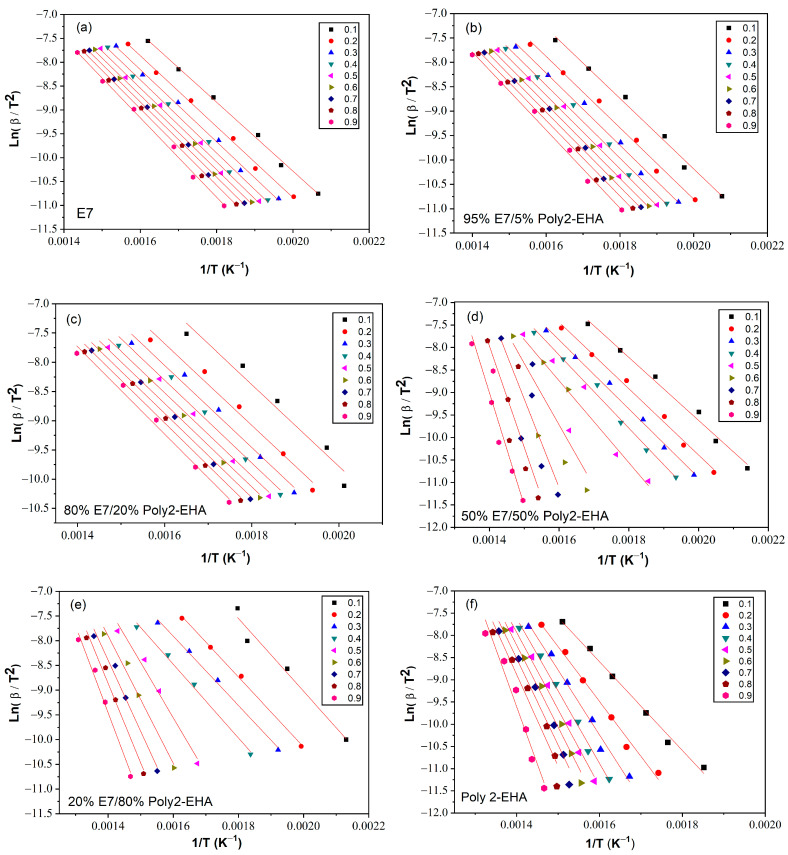
*Ln*(*β*/*T*^2^) as function of 1/*T* for Poly(2-EHA)/E7 mixtures, according to the conversion *α*, using the approach of KAS et al. [63]. (**a**) 100 wt-% E7, (**b**) 95 wt-% E7, (**c**) 80 wt-% E7, (**d**) 50 wt-% E7, (**e**) 20 wt-% E7 and (**f**) Poly(2-EHA).

The determination of the activation energy allows to estimate the number of steps involved in a reaction and the nature of the processes involved [62]. The variation of the activation energy as a function of the degree of conversion *α* was studied using the iso-conversion methods presented in Section 3. The kinetic parameters obtained by the meth-ods of Tang, FWO and KAS were calculated according to Equation (5), (6) and (7), respectively, for values of conversion *α* from 0.1 to 0.9. To determine the kinetic parameters, the same value of *α* was chosen for all curves with different heating rates. Figure 6 shows a representation of $\ln\left(\frac{\beta }{T_{\alpha i}^{1.894661}}\right)$ versus 1/T, according to the Tang method, for different conversion values. The corresponding results from the FWO approach are shown in Figure 7, where lnβ is given as a function of 1/T. Finally, Figure 8 shows plots of $ln\frac{\beta_{i}}{T_{\alpha i}^{2}}$ as a function of 1/T, corresponding to the KAS method. The curves obtained by these three methods represent a linear behavior for all compositions studied, applying different heating rates. The *E_α_* values were calculated from the slopes of these lines.

Table 1 summarizes the results of the thermal activation energies calculated from the three theoretical models for the poly(2-EHA)/E7 system. It can be seen that the global evolution of these activation energies was the same for the three models. The coefficient of determination *R*^2^ presented values generally higher than 0.97. However, the 50 wt-% mixture presented lower *R*^2^ data because two degradation processes were observed, that of the LC and that of the poly(2-EHA).

**Figure 9:**
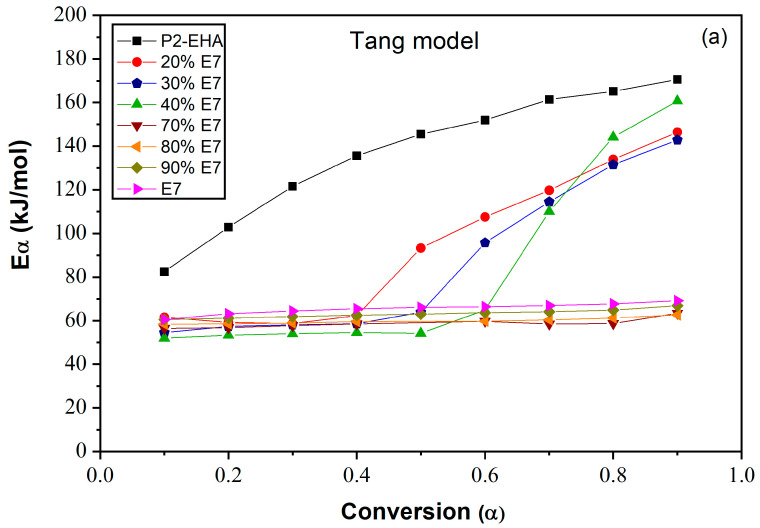

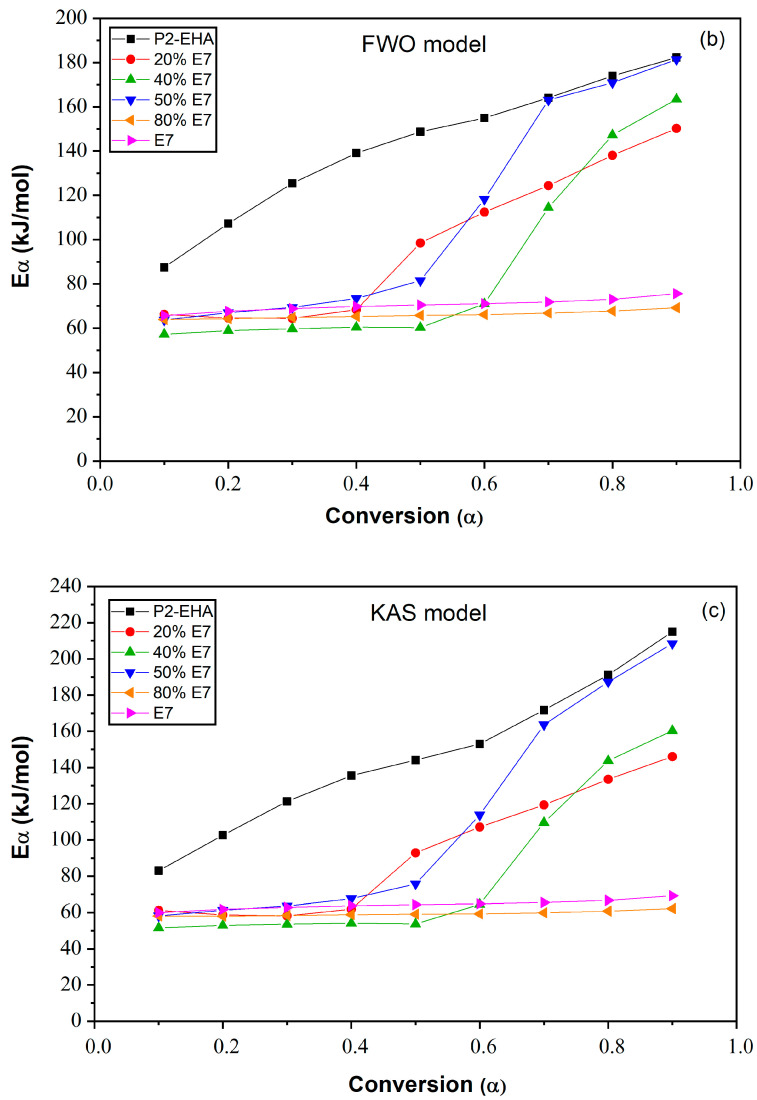
Variation of the apparent activation energies of E7 and Poly(2-EHA) and their mixtures according to the conversion α: (**a**) Tang model, (**b**) FWO model and (**c**) KAS model.

Figure 9a, b and c shows the plots of *E_α_* of the Poly 2-EHA/E7 mixtures as a function of conversion *α*, determined by the Tang, FWO and KAS methods, respectively. The *E_α_* value for the LC E7 remains constant at 64 kJ/mol for all conversions *α*, for the three models. For the polymer Poly(2-EHA), using Tang and FWO models, the activation energy shows a variation ranging from 80 kJ/mol, for conversion *α* = 0.1, to 170 kJ/mol for *α* = 0.9. For the third model (KAS), this energy varies between 80 and 220 kJ/mol in the same range of *α*. In the case of photochemically crosslinked Poly(2-EHA), Merah et al. [43] observed a single degradation step, characterized by an activation energy estimated at 293 kJ/mol. This energy corresponds to the entire thermal decomposition of Poly(2-EHA), which mainly produces monomers through a so-called unzipping process. For LC-rich mixtures, the activation energy remains constant with the conversion so that the obtained values were practically identical to those of pure LC. In these blends (above 50 wt-% E7), the LCs phase separate to form macroscopic domains and thus determine the total activation energy. In the case of polymer-rich mixtures, the activation energy remains constant over the conversion interval from 0.1 to 0.6. Beyond *α* = 0.6, using the method of Tang et al., it increases to values between 140 and 160 kJ/mol. These values change slightly using the other two methods:

In the case of the FWO model, *E_α_* varies between 80 and 180 kJ/mol, while for the KAS method, values between 80 and 200 kJ/mol were obtained.

**Figure 10:**
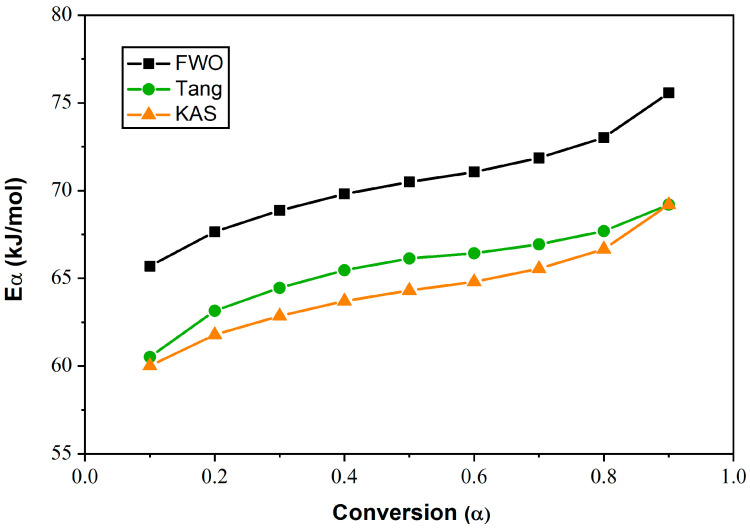
Dependence of *E_α_* on conversion *α* for E7, evaluated by Tang, FWO and KAS methods.

Figure 10 shows the dependence of *E_α_* on *α* for the LC E7. The activation energy increases with the degree of conversion regardless of the isoconversion method used. The *E_α_* values obtained from the Tang method were found to be close to those collected from the KAS method, varying between 60 kJ/mol and 69 kJ/mol. For the FWO model, this energy increases from 65 kJ/mol to 75 kJ/mol with increasing *α*. For a given value of *α*, *E*_FWO_ > *E*_Tang_ ≈ *E*_KAS_.

## 5. Conclusions

The thermal degradation kinetics and stability of a model polymer/LC system were investigated by TGA. The system investigated in this report consists of linear poly(2-EHA) and the eutectic nematic LC mixture E7. Different heating rates were used for TGA under nitrogen atmosphere. For poly(2-EHA)/E7 blends containing high concentrations of E7, the TGA results showed a thermal behavior similar to that of pure E7. In this case, a single degradation temperature corresponding to E7 was observed, which increases with the concentration of LC. For lower E7 content, two degradation temperatures were observed, one corresponding to poly(2-EHA) and the other to LC. For polymer-rich mixtures, a slight variation in the degradation temperatures of E7 and polymer was observed.

The results obtained by TGA were rationalized by isoconversion methods to derive kinetic parameters. In particular, *E_α_* was calculated at different conversion rates (*α*), using theoretical methods developed by Tang, FWO and KAS. Good agreement between all the theories and experimental data was obtained. The coefficient of determination *R*^2^ of the linear relationships presented values generally higher than 0.97.

## Figures and Tables

**Table 1:** Activation energies and regression parameters *R*^2^ for Poly(2-EHA)/E7 mixtures. *R*^2^ represents a goodness-of-fit measure for linear regression.

Composition	Conversion	Tang Model		FWO Model		Kissinger Model	
		*E_α_* (kJ/mol)	*R* ^2^	*E_α_* (kJ/mol)	*R* ^2^	*E_α_* (kJ/mol)	*R* ^2^
E7	0.1 0.2 0.3 0.4 0.5 0.6 0.7 0.8 0.9	60.52 63.15 64.46 65.46 66.13 66.43 66.93 67.69 69.19	0.9933 0.9927 0.9918 0.9899 0.9900 0.9895 0.9907 0.9907 0.9912	65.69 67.66 68.86 69.80 70.49 71.06 71.86 73.01 75.56	0.9964 0.9960 0.9958 0.9951 0.9951 0.9951 0.9960 0.9966 0.9973	60.01 61.78 62.85 63.70 64.30 64.80 65.55 66.66 69.19	0.9962 0.9955 0.9948 0.9936 0.9936 0.9935 0.9945 0.9947 0.9950
95% E7	0.1 0.2 0.3 0.4 0.5 0.6 0.7 0.8 0.9	60.48 61.18 61.85 62.48 63.00 63.65 64.07 64.88 66.89	0.9905 0.9906 0.9906 0.9903 0.9903 0.9905 0.9899 0.9906 0.9909	65.74 66.73 67.57 68.32 68.94 69.67 70.18 71.06 73.10	0.9931 0.9931 0.9932 0.9930 0.9929 0.9930 0.9927 0.9932 0.9934	60.10 60.77 61.43 62.05 62.57 63.21 63.63 64.43 66.43	0.9903 0.9904 0.9905 0.9901 0.9901 0.9903 0.9898 0.9904 0.9907
80%E7	0.1 0.2 0.3 0.4 0.5 0.6 0.7 0.8 0.9	58.4 58.47 58.85 59.55 59.76 60.37 61.26 62.62 63.85	0.9484 0.9736 0.9784 0.9786 0.9800 0.9821 0.9831 0.9850 0.9860	63.93 64.25 64.83 65.28 65.79 66.10 66.80 67.76 69.19	0.9618 0.9814 0.9850 0.9851 0.9862 0.9877 0.9884 0.9898 0.9905	58.17 58.05 58.42 58.85 59.11 59.30 59.91 60.80 62.16	0.9857 0.9847 0.9827 0.9817 0.9796 0.9783 0.9779 0.9731 0.9512
50% E7	0.1 0.2 0.3 0.4 0.5 0.6 0.7 0.8 0.9	58.27 61.37 63.66 67.64 75.80 146.88 163.73 187.37 208.59	0.9873 0.9907 0.9924 0.9883 0.8292 0.8643 0.5535 0.6083 0.8893	63.66 66.99 69.42 73.42 81.45 118.28 166.07 188.88 209.38	0.9910 0.9935 0.9947 0.9917 0.8617 0.5453 0.5900 0.6391 0.8998	58.20 61.29 63.58 67.56 75.72 113.84 163.65 187.29 208.50	0.8892 0.6081 0.5532 0.4926 0.8289 0.9887 0.9924 0.9906 0.9873
20% E7	0.1 0.2 0.3 0.4 0.5 0.6 0.7 0.8 0.9	61.54 59.14 58.62 62.30 93.29 107.54 119.80 133.95 146.45	0.9582 0.9964 0.995 0.9921 0.9724 0.9752 0.9761 0.9774 0.9816	66.24 64.60 64.44 68.34 98.49 112.43 124.39 138.09 150.24	0.9671 0.9977 0.9968 0.9948 0.9783 0.9799 0.9803 0.9811 0.9845	61.19 58.74 58.20 61.86 92.86 107.11 119.37 133.53 146.03	0.9815 0.9771 0.9758 0.9749 0.9720 0.9919 0.9948 0.9963 0.9577
Poly(2-EHA)	0.1 0.2 0.3 0.4 0.5 0.6 0.7 0.8 0.9	82.50 102.87 121.60 135.64 145.50 151.96 161.41 165.16 170.64	0.9959 0.9788 0.9865 0.9921 0.9608 0.7164 0.9476 0.9825 0.9598	91.95 112.86 131.94 146.23 156.38 162.93 172.55 180.93 223.22	0.9964 0.9819 0.9884 0.9932 0.9909 0.9799 0.9541 0.9359 0.9640	82.97 102.64 121.27 135.56 144.12 153.05 171.74 191.21 214.99	0.9857 0.9847 0.9827 0.9817 0.9796 0.9783 0.9779 0.9731 0.9512

## Data Availability

Data set presented in this study is available in this article.
